# Role of non-coding RNAs and exosomal non-coding RNAs in retinoblastoma progression

**DOI:** 10.3389/fcell.2022.1065837

**Published:** 2022-12-23

**Authors:** Nasrin Ahangar Davoodi, Sajad Najafi, Zari Naderi Ghale-Noie, Ashkan Piranviseh, Samaneh Mollazadeh, Sahar Ahmadi Asouri, Zatollah Asemi, Mohammadamin Morshedi, Seyed Saeed Tamehri Zadeh, Michael R. Hamblin, Amirhossein Sheida, Hamed Mirzaei

**Affiliations:** ^1^ Eye Research Center, Rassoul Akram Hospital, Tehran University of Medical Sciences, Tehran, Iran; ^2^ Department of Medical Biotechnology, School of Advanced Technologies in Medicine, Shahid Beheshti University of Medical Sciences, Tehran, Iran; ^3^ Department of Medical Genetics, Faculty of Medicine, Mashhad University of Medical Sciences, Mashhad, Iran; ^4^ Brain and Spinal Cord Injury Research Center, Tehran University of Medical Sciences, Tehran, Iran; ^5^ Natural Products and Medicinal Plants Research Center, North Khorasan University of Medical Sciences, Bojnurd, Iran; ^6^ Research Center for Biochemistry and Nutrition in Metabolic Diseases, Institute for Basic Sciences, Kashan University of Medical Sciences, Kashan, Iran; ^7^ Student Research Committee, Kashan University of Medical Sciences, Kashan, Iran; ^8^ School of Medicine, Kashan University of Medical Sciences, Kashan, Iran; ^9^ School of Medicine, Tehran University of Medical Sciences, Tehran, Iran; ^10^ Laser Research Centre, Faculty of Health Science, University of Johannesburg, Doornfontein, South Africa

**Keywords:** Retinoblastma, non-coding RNAs, exosome, microRNA, circular RNA

## Abstract

Retinoblastoma (RB) is a rare aggressive intraocular malignancy of childhood that has the potential to affect vision, and can even be fatal in some children. While the tumor can be controlled efficiently at early stages, metastatic tumors lead to high mortality. Non-coding RNAs (ncRNAs) are implicated in a number of physiological cellular process, including differentiation, proliferation, migration, and invasion, The deregulation of ncRNAs is correlated with several diseases, particularly cancer. ncRNAs are categorized into two main groups based on their length, i.e. short and long ncRNAs. Moreover, ncRNA deregulation has been demonstrated to play a role in the pathogenesis and development of RB. Several ncRNAs, such as miR-491-3p, miR-613,and SUSD2 have been found to act as tumor suppressor genes in RB, but other ncRNAs, such as circ-E2F3, NEAT1, and TUG1 act as tumor promoter genes. Understanding the regulatory mechanisms of ncRNAs can provide new opportunities for RB therapy. In the present review, we discuss the functional roles of the most important ncRNAs in RB, their interaction with the genes responsible for RB initiation and progression, and possible future clinical applications as diagnostic and prognostic tools or as therapeutic targets.

## 1 Introduction

Retinoblastoma (RB) accounts for 3% of all pediatric malignancies, and is the most prevalent intraocular malignant tumor (Rao and Honavar, 2017; Dimaras and Corson, 2019). There are no racial, regional, or gender differences in the incidence of retinoblastoma, which is roughly 1/15,000 across the world (Maheshwari and Finger, 2018). For unilateral retinoblastoma, the average age of diagnosis is 24 months, compared to 12 months for bilateral retinoblastoma. Bilateral tumors occur in between 30% and 40% of retinoblastoma cases. Most cases have been reported to be sporadic (94%), while only 6% of new cases were determined to be familial (Bishop and Madson, 1975; Maheshwari and Finger, 2018). This malignant tumor accounts for 1% of all infant deaths, and can be extremely malignant owing to intracranial metastasis (He et al., 2014). Retinoblastoma typically progresses rapidly without treatment, and damages the structure of the eyeball resulting in blindness (Aerts et al., 2015). Moreover, the tumor can directly invade the brain *via* the optic nerve, or spread to other parts of body *via* the blood, including bones, lungs, and other distant organs (Correa-Acosta et al., 2018). It imposes a heavy emotional, financial, and medical burden on patients and society, as well as affecting their long-term health and the quality of life. The survival rate of patients with retinoblastoma is low, despite some recent significant advances in treatment. One reason is that early disease detection seems to be difficult to achieve (Park et al., 2014). Hence, early diagnosis and prompt treatment are crucial in order to prevent loss of vision and metastasis of retinoblastoma. Around 75% of the entire human genome can be transcribed into RNAs, although only 3% can be transcribed into protein-coding mRNAs (Kimura, 2020). Non-coding RNAs (ncRNAs) are classified into distinct types based on their length, structure, and location. The three main types of ncRNA are, circular RNAs (circRNA), long ncRNAs (lncRNA), and microRNAs (miRNA). MiRNAs are small RNAs with a typical length of 22 nucleotides (nt). After binding of miRNAs to the complementary base sequence in the target mRNAs, they are is degraded by the RNA-induced silencing complex (RISC) (Vos et al., 2019). LncRNAs and circRNAs are both longer than 200 nt, however circRNAs are closed circles whereas lncRNAs are linear. LncRNAs and circRNAs can both arise by transcription of exons, introns, intergenic regions, or 5′/3′-untranslated regions of various genes. However, they can fold to produce complex secondary structures which can interact with proteins, DNA, or RNA (Invalid Citationa). LncRNAs and circRNAs can regulate expression of genes *via* various mechanisms. To prevent the targeted mRNA from being degraded, they can act as miRNA decoys or sponges. Furthermore, they can alter the affinity of transcription factors for binding to their promoters, in order to regulate the expression of specific genes (Zhao et al., 2014). In the present review, we discuss the role of various non-coding RNAs in retinoblastoma.

## 2 MicroRNAs and retinoblastoma

MicroRNAs (miRNAs) are a group of endogenous non-coding small RNAs with the ability to regulate the expression of their target genes (Lytle et al., 2007). They directly interact with the 3′-untranslated region of mRNA, and then negatively affect the expression of the target genes, either by mRNA degradation or suppression of translation (Guarnieri and DiLeone, 2008). Based on the miRBase database, the human genome includes 4,469 genes which encode miRNAs, consisting of 1,881 precursor and 2,588 mature miRNAs. Various types of miRNAs have been found to be abnormally expressed in RB samples. These altered miRNAs can affect many cancer-related processes, including the epithelial-mesenchymal transition, cell proliferation and survival, apoptosis, metastasis, and angiogenesis (Delsin et al., 2019). MiR-98 (Reese et al., 2005) and miR-186 (Davidson et al., 2004) are two examples of miRNAs that are overexpressed in RB and contribute to the malignancy of the tumor, whereas miR-98 (Reese et al., 2005), miR-186(18), and miR-106b (Reese et al., 2006) are expressed only at low levels in RB and are therefore expected to act as tumor inhibitors.

Stannin (SNN), which has a single transmembrane helix, an unstructured linker domain, and a cytoplasmic domain, is a highly conserved protein in vertebrate animals. It has previously been reported to be strongly associated with the toxicity of trimethyltin (TMT) and has a significant effect on apoptosis (Reese et al., 2005). It has been demonstrated that SNN plays a critical role in the toxic effect of organotin (Davidson et al., 2004) and endosomal maturation (Pueyo et al., 2016). Many investigations have also suggested the role of SNN in tumor growth (Reese et al., 2006). In a study by Reese et al. it was demonstrated that protein kinase C could regulate tumor necrosis factor-alpha (TNF-α) leading to increased expression of SNN (Reese et al., 2006). SNN plays a crucial role in MAPK signaling pathways (Huang et al., 2016), and the MAPK signaling pathway is connected to a number of cancers. The expression of SNN may be affected by certain miRNAs. The effects of miR-491-3p on proliferation, migration, invasion, and apoptosis in RB cells was examined in a study by Hu et al. (Hu et al., 2021). Primary RB tissue samples from 15 patients were collected as well as paired adjacent non-cancerous tissues. Quantitative real-time PCR (qRT-PCR) was used to examine the expression profile of miR-491-3p. In order to investigate the expression profiles of epithelial–mesenchymal transition (EMT)-related proteins (vimentin, E-cadherin, and N-cadherin) in RB tissues and RB cell lines they performed qRT-PCR, Western blotting and *in situ* immunocytochemistry. Cell proliferation was measured using MTS and colony formation assays. Apoptosis was determined using FACS, while cell migration and invasion were examined using transwell chambers. Target gene prediction databases were used to predict the genes that miR-491-3p would bind to. To evaluate the relationship between miR-491-3p and SNN, dual luciferase reporter assays were carried out. In 15 pairs of Rb tissues as well as RB cell lines it was found that miR-491-3p was noticeably downregulated.

Artificial overexpression of miR-491-3p dramatically suppressed RB cell proliferation, migration, and invasion, while it increased apoptosis. By contrast, treatment with a miR-491-3p inhibitor produced the opposite results, leading to less apoptosis, and increased proliferation in ARPE-19 cells. Additionally, miR-491-3p altered the morphology of RB cell lines resulting in cells that were more adherent, and it significantly reduced the expression of vimentin and N-cadherin while increasing E-cadherin suggesting it could inhibit the EMT. The SNN was identified as a miR-491-3p base-pairing target, and the two molecules could interact with each other. They also discovered that the overexpression of SNN almost completely neutralized the effects of miR-491-3p in RB cells. In total, their findings suggested that miR-491-3p acts as a tumor suppressor gene in RB, and could inhibit the growth and metastasis of tumors as a novel therapeutic target in RB (Hu et al., 2021).

Wnt-inhibitory factor-1 (WIF1) acts as a secreted repressor protein that can directly bind to various ligands of the Wnt signaling pathway, and inhibit their activity (Hsieh et al., 1999; Poggi et al., 2018). In previous studies, WIF1 was shown to be involved in a variety of biological processes including tumor growth, cell cycle, metastasis, apoptosis, and stemness (Wu et al., 2012a; Ramachandran et al., 2012; Ramachandran et al., 2014; Wang et al., 2015; Huang et al., 2016). In numerous human cancers, WIF1 promoter hypermethylation was linked to a poor prognosis (Lee et al., 2013; Roperch et al., 2013; Zhao et al., 2017a; Lin et al., 2017; Zhou et al., 2018). Additionally, a number of ncRNAs such as HOTAIR, HOXC6, miR-181a, miR-552, miR-590-3p, and miR-603, have been shown to regulate WIF1 expression at the transcriptional level (Ji et al., 2014; Guo et al., 2015; Jiang et al., 2016; Feng et al., 2017a; Li et al., 2018; Yan et al., 2018). Gastric tumors have been linked to hypermethylation of the WIF1 promoter, which inhibits its expression and abnormally activates the Wnt signaling pathway (Poggi et al., 2018). Moreover, WIF1 hypermethylation has been shown to be correlated with poor survival rate in some cancer patients, such as non-small-cell lung cancer (Guo et al., 2017), esophageal squamous cell carcinoma (Guo et al., 2016), and chondrosarcoma (Liu et al., 2017).

Using the GEO datasets, Li et al. set up an interaction network involving numerous distinct genes and miRNAs that were selectively expressed in retinoblastoma (Fuchs et al., 2001). They found that RB cell lines and tissues highly expressed miR-340, and up-regulation of miR-340 was associated with a poor prognosis in patients. The Gene Expression Omnibus (GEO) repository was used to retrieve differentially expressed miRNAs (DEmiRs) and genes (DEGs) in retinoblastoma. Besides, qRT-PCR was used to determine the expression of WIF1 and miR-340 in RB tissues and cell lines. Gain-of-function and loss-of-function assays were used to examine the effects of miR-340 on cell proliferation, migration, and invasion. Bioinformatics analysis and luciferase reporter experimental assays were used to investigate the interaction between miR-340 and WIF. Blood and tissue samples from retinoblastoma patients displayed a total of 11 DEmiRs. They confirmed that miR-340 was the most abundantly expressed miRNA, and was associated with ICRB stage, tumor size, and optic nerve invasion. They proposed that miR-340 could promote RB cell proliferation, migration, and invasion. They subsequently set up a regulatory network of miRNA-mRNA pairs after identifying 26 DEGs from three retinoblastoma GEO datasets. Further research revealed that miR-340 directly targeted WIF1. Furthermore, WIF1 overexpression might inhibit retinoblastoma progression induced by miR-340 *in vitro* and *in vivo*. Overall, miR-340 modulates WIF1 and then acts as an oncomiRNA to enhance RB cell proliferation, migration, and invasion. They also identified multiple miRNAs and genes that could help researchers better understand retinoblastoma pathogenesis (Fuchs et al., 2001).

E2F5 is a transcription factor and a member of the E2 promoter binding factor (E2F) family, involved in cell proliferation (Dimova and Dyson, 2005; Cao et al., 2010; Wan et al., 2010). Basically, E2F5 links the cell cycle to post-transcriptional pathways, so it is essential for regulating cell growth, and also affects biological processes involved in cancer development (Ishimoto et al., 2013; Xie et al., 2020). Inhibition of E2F5 may be able to reduce metastasis and growth of gastric cancer, pointing to a possible role for this protein in new treatments (Yao and Yan, 2018). E2F5 may be involved in the development of other cancers, such as hepatocellular carcinoma, colon, breast, ovarian, osteogenic sarcoma, and esophageal squamous cell carcinoma (Fuchs et al., 2001; Lassmann et al., 2007; Umemura et al., 2009; Kothandaraman et al., 2010; Jiang et al., 2011). E2F5 expression was found to be increased in RB where it affected the cell cycle, growth and proliferation (Zhang et al., 2017a).

Zhang et al. measured the expression of miR-613 in human RB samples and investigated its function in RB cells (Zhang et al., 2017a). They found that the level of miR-613 was lower in RB tissues and cell lines. *In vitro* studies showed that overexpression of miR-613 led to suppression of RB cell proliferation, migration, and invasion, as well as producing cell cycle arrest. Besides, miR-613 overexpression prevented retinoblastoma cells from developing tumors *in vivo*. They also showed that E2F5 was a direct target of miR-613. The introduction of E2F5 mRNA without a 3′-untranslated region abrogated the effects miR-613 on proliferation and invasion. Their findings showed that miR-613 acts as a tumor suppressor in retinoblastoma by downregulating E2F5. Their data supported the idea of targeting the miR-613/E2F5 axis as a potential therapeutic approach for retinoblastoma (Table 1) (Zhang et al., 2017a).

**TABLE 1:** The role of miRNAs in retinoblastoma initiation and progression.

MicroRNA	Expression status	Target	Detection method	Effect	Model	Type of cell line	Reference
miR-129	Down	MDM2/4	qPCR	Increased protein levels of p53, p21, Bax, and cleaved-caspase 3, decreased Bcl-2 protein	Human^1^, *in vitro*	WERI-RB1, ARPE-19, Y79	Yao et al. (2021)
miR-153	Down	IGF1R	RT-qPCR	Inhibited RB cell proliferation, invasion and migration, and promoted apoptosis	Human, *in vitro*	WERI-RB1, Y79	Guo et al. (2021)
miR-214-3p	Down	ABCB1, XIAP	qRT-PCR	Promoted apoptosis	Human, *in vitro*, *in vivo*	WERI-RB1, SO-RB50, Y79, ARPE-19, HEK-293T	Yang et al. (2020a)
miR-491-3p	Down	SNN	qRT-PCR	Enhanced apoptosis, suppressed proliferation, migration and invasion	Human, *in vitro*	ARPE-19, WERI-RB1, Y79	Hu et al. (2021)
miR-192	Down	Integrin Alpha 1	Nanofluorescence	Decreased viability and invasion of RB cell line, increased apoptosis	*In vitro*	RB116	Gao et al. (2021a)
miR-9	Down	PTEN	qRT-PCR	Suppressed cell viability, proliferation and migration, inhibited tumor formation	Human, *In vitro*, *In vivo*	HXO-Rb44, Y79, SO-RB50, ARPE-19	Gao et al. (2021b)
miR-144	Down	−	RT-PCR	−	Human	−	Zheng et al. (2020)
miR-449a, miR-449b	Up	−	RT-PCR	Inhibited proliferation, increased apoptosis	Human, *in vitro*	Y79, WERI-RB1	Martin et al. (2013)
miR-142-5p	Up	PTEN	qRT-PCR	Promoted proliferation, migration and invasion	Human, *in vitro*	WERI-RB1, Y79	Zhang and Zheng, (2021)
	Down	MYCN	RT-qPCR	Inhibited proliferation, migration, invasion, and increased apoptosis	Human, *in vitro*	Y79, WERI-RB1, ARPE-19	Li et al. (2021a)
miR-340	Up	WIF1	qRT-PCR	Promoted proliferation, migration and Invasion	Human, *in vitro*, *in vivo*	WERI-RB1, SO-RB50, Y79, ARPE-19	Li et al. (2021b)
	Down	KIF14	qRT-PCR	Decreased proliferation, induced apoptosis	*In vitro*	Y79, WERI-RB1, ACBRI-181	Xu et al. (2021a)
miR-138	Up	RARα	RT-PCR	Increased viability	Human, *in vitro*	RB355, Y79, WERI-RB1	Busch et al. (2021)
miR-4319	Down	EMMPRI/CD147	RT-qPCR	Inhibited proliferation, migration, invasion	Human, *in vitro*	HXO-RB44, WERI-RB1, SO-RB50, RB-Y79, FMC-RB	Wu et al. (2021)
MiR-613	Down	E2F5	qRT-PCR	Suppressed proliferation, invasion, and tumor formation	Human, *in vitro*	SO-RB50, Y79, WERI-RB1, ARPE-19	Zhang et al. (2017a)
miR-31, miR-200a	Down	−	RT-PCR	Inhibited proliferation	Human, *in vitro*	Y79, Weri1	Montoya et al. (2015)
miR-330	Down	ROCK1	RT-qPCR	Reduced viability and invasion	Human, *in vitro*	Y79, WERI-RB1, SO-RB50, ARPE-19	Wang et al. (2019a)
miR-34a	Down	Notch1	RT-qPCR	Inhibited proliferation and increased the chemosensitivity of RB cells	*In vitro*	ACBRI-181, HXO-RB44, Y79	(INVALID CITATIONa)
	Down	MAGE-A	RT-qPCR	Promoted viability, decreased apoptosis	*In vitro*, human	HXO-Rb44, SO-Rb50, Y79, WERI-RB1	Yang et al. (2019a)
miR-140-5p	Down	CEMIP, CADM3	qRT-PCR	Inhibited proliferation, migration, and invasion	Human, *in vitro*	Y79, WERI-RB1, 293T	Miao et al. (2018)
miR-101-3p	Down	EZH2, HDAC9	RT-qPCR	Inhibited proliferation, viability, caused cell cycle arrest	Human, *in vitro*	Y79, WERI-RB1	Jin et al. (2018)
miR-204-3p	Down	Wnt	qRT-PCR	Inhibited proliferation, invasion, migration	*in vitro*, *in vivo*	HXO-RB44, Y79, WERI-RB1, ARPE-19	Sun et al. (2020a)
miR-486-3p	Down	ECM1	qRT-PCR	Inhibited proliferation, invasion, migration	Human, *in vitro*	ARPE-19, Y79, SO-RB50, WERI-RB1	Yang et al. (2020b)
miR-520a-3p	Down	EGFR	RT-qPCR	Decreased proliferation, increased apoptosis	Human, *in vitro*	WERI-RB1, Y79, SO-RB50, SO-RB70, ARPE-19	Xia et al. (2019a)
miR-504	Down	AEG-1	RT-qPCR	Inhibited proliferation and invasion	Human, *in vitro*	SO-RB50, Y79, WERI-RB1, ARPE-19	Wang et al. (2019b)
miR-598	Down	E2F1	qRT‐PCR	Inhibited viability and metastasis	Human, *in vitro*	ARPE‐19, WERI‐RB1, Y79	Liu et al. (2020a)
miR-433	Down	Notch1, PAX6	qRT-PCR	Inhibited proliferation, migration and invasion, and induced cell cycle arrest and apoptosis	Human, *in vitro*	Y79, WERI-RB1	Li et al. (2016a)
miR-145	Down	ABCE1	qRT-PCR	Inhibited viability and proliferation, and induced apoptosis	*In vitro*	HEK-293T, Y79	Wei et al. (2017)
	Down	ADAM19	qRT-PCR	Suppressed cell proliferation, migration, invasion	Human, *in vitro*	Y79, WERI-RB1, SO-RB50	Sun et al. (2015)
miR-936	Down	HDAC9	RT-qPCR	Reduced proliferation, colony formation, migration, and invasion, increased apoptosis	Human, *in vitro*	Y79, WERI-RB1, SO-RB50, APRE-19	Xu et al. (2020a)
miR-125a-5p	Down	TAZ	qRT-PCR	Suppressed tumor development and cell proliferation	Human, *in vitro*	Y79, WERI-RB1	Zhang et al. (2016b)
miR-338-3p	Down	NOVA1	RT-qPCR	Inhibited proliferation, migration and invasion, and promoted apoptosis	Human, *in vitro*	HXO-RB44, SO-RB50, Y79, WERI-RB1, ARPE-19	Sun et al. (2021)
miR-874	Down	MTDH	RT-qPCR	Inhibited proliferation and invasion	Human, *in vitro*	ARPE-19, SO-RB50, Y79, WERI-RB1	Zhang et al. (2018a)
miR-98	Down	IGF1R	RT-qPCR	Inhibited proliferation, invasion and migration	Human, *in vitro*	WERI-RB1, Y79, SO-RB50, ARPE-19	Guo et al. (2019)
miR-221/222	Up	-	qRT-PCR	Promoted proliferation, migration, and invasion	Human, *in vitro*	Y79	Liu et al. (2018a)
miR-363-3p	Down	PIK3CA	RT-qPCR	Inhibited proliferation, induced apoptosis	Human, *in vitro*	WERI-RB1	Ma et al. (2020)
miR-154-5p	-	ATG7	qRT-PCR	Inhibited proliferation and migration, induced apoptosis	*In vitro*, *in vivo*	Y79	Liu et al. (2020b)
miR-382	Down	BDNF	RT-qPCR	Inhibited proliferation and invasion	Human, *in vitro*	Y79, WERI-RB1, SO-RB50	Song et al. (2017)
miR-492	Up	LATS2	RT-qPCR	Increased proliferation and invasion	Human, *in vitro*	WERI-RB1, SO-RB50, Y79, ARPE-19	(INVALID CITATIONa)
miR-182	Up	CADM2	RT-qPCR	Increased viability, invasion, angiogenesis	Human, *In vitro*, *in vivo*	Y79, WERI-RB1	Huang et al. (2018a)
miR-204	Down	CyclinD2, MMP-9	qRT-PCR	Inhibited proliferation, migration and invasion	Human, *in vitro*, *in vivo*	SO-RB50	Wu et al. (2015)
miR-198	Up	PTEN	RT-qPCR	Increased proliferation and invasion, regulated PI3K/AKT signaling pathway	Human, *in vitro*	Y79, SO-RB50, WERI-RB1, ARPE-19	(INVALID CITATIONa)
miR-320	Down	SP1	RT-qPCR	Inhibited proliferation, migration and invasion	Human, *in vitro*	Y79, WERI-RB1, SO-RB50, HEK293T	Zhao et al. (2017b)
	Up	HIF-1α	RT-qPCR	Promoted autophagy	*In vitro*, human	WERI-RB1	Liang et al. (2017)
miR-34A	Up	HMGB1	qRT-PCR	Induced apoptosis, increased CASP3activity, inhibited autophagy	*In vitro*	Y79, WERI-RB1, HCT116	Liu et al. (2014)
miR-129-5p	Down	PAX6	RT-qPCR	Inhibited proliferation, migration and invasion, *via* PI3K/AKT signaling pathway	*In vivo*, *in vitro*	AC-BRI-181, Y79, WERI-RB1	Liu et al. (2019a)
miR-125b	Up	DRAM2	RT-PCR	Increased proliferation and migration	Human, *in vitro*	HXO-Rb44, Y79, SO-RB50, ARPE-19	Bai et al. (2016)
miR-497	Down	VEGFA	RT-qPCR	Inhibited proliferation, migration and invasion	Human, *in vitro*	Y79, WERI-RB1	Li et al. (2017)
miR-361-5p	Down	CLDN8	qRT-PCR	Inhibited proliferation, increased apoptosis	*In vitro*	Y79, SO-RB50, ARPE-19	Liu et al. (2019b)
miR-362-3p	Down	USP22	RT-qPCR	Inhibited proliferation, migration and invasion, induced apoptosis	Human, *in vitro*	ARPE-19, WERI-RB1, Y79	Rong et al. (2021)
miR-655	Down	PAX6	RT-qPCR	Inhibited proliferation and invasion, increased apoptosis	Human, *in vitro*	Y79, SO-RB50, WERI-RB1	Zhang et al. (2018b)
miR-498	Up	CCPG1	qRT-PCR	Promoted proliferation, inhibited apoptosis	*In vitro*	Y79, ARPE-19	Yang et al. (2018)
miR-146a	Down	NOVA1	RT-PCR	Inhibited viability, proliferation and invasion, increased apoptosis	Human, *in vitro*	Y79, WERI-Rb-1	Liu et al. (2021a)
miR-21	Up	-	RT-PCR	Increased proliferation, migration and colony formation	Human, *in vitro*	HXO-RB44	Ding et al. (2014)
	Up	PTEN	qRT-PCR	Promoted proliferation, migration, invasion, suppressed apoptosis	*In vitro*, human	WERI-RB1	Gui et al. (2016)
miR-137	Down	COX-2	RT-qPCR	Inhibited proliferation and invasion	Human, *in vitro*	WERI-RB1, Y79, SO-RB50, ARPE-19	Zhang et al. (2018c)
miR-224-3p	Up	LATS2	RT-qPCR	Inhibited apoptosis, increased proliferation and angiogenesis	Human, *in vitro*, *in vivo*	Y79	Song et al. (2020)
miR-375	Down	ERBB2	RT-PCR	Inhibited proliferation, migration and invasion, suppressed tumor progression *via* inhibiting MAPK1/MAPK3 signalling pathway	Human, *in vitro*	Y79, SO-RB50, ARPE-19	Liu et al. (2022)
miR-25-3p	Up	PTEN	qRT-PCR	Increased proliferation, colony formation, anchorage-independent growth, cell migration and invasion	Human, *in vitro*, *in vivo*	ARPE-19, WERI-RB1, SO-RB50, Y79, HEK293 T	Wan et al. (2019)
miR-181a-5p	Down	NRAS	qRT-PCR	Reduced proliferation, migration, and invasion, enhanced apoptosis	Human, *in vitro*	ARPE-19, HXO-RB44, SO-Rb50, WERI-RB1, Y79	Ouyang et al. (2022)
miR-186	Down	DIXDC1	RT-qPCR	Inhibited proliferation, invasion, and Wnt signaling in cells	Human, *in vitro*	Y79, WERI-RB1, SORB50, ARPE-19	Che et al. (2018)
	Down	ATAD2	RT‐qPCR	Reduced viability, invasion, migration and angiogenesis, promoted apoptosis	*In vitro*, human	HXO‐Rb44, ACBRI‐181	Wu et al. (2019a)
miR-106b	Up	Runx3	RT-PCR	Increased viability, proliferation and migration, decreased apoptosis	*In vitro*	Y79	Yang et al. (2017)
miR-29a	Down	STAT3	qRT-PCR	Inhibited proliferation, migration, and invasion, promoted apoptosis	Human, *in vitro*	Y79, SO-RB50	Liu et al. (2018b)
miR-758	Down	PAX6	RT-qPCR	Inhibited proliferation, migration and invasion, induced apoptosis	Human, *in vitro*	Y79, WERI-RB1, SO-RB50, ARPE-19	(INVALID CITATIONa)
miR-141-3p	Up	SUSD2	qRT-PCR	Increased proliferation and angiogenesis	Human, *in vitro*	HUVECs, Y79, WERI-RB1, ARPE-19	Liu and Wen, (2022)
miR-494	Up	PTEN	qRT-PCR	Increased proliferation invasion and migration	*In vitro*, human	Y79, SO-RB50, APRE-19	Xu et al. (2020b)
miR-143	Down	−	RT-PCR	Suppressed viability and invasion, increased apoptosis	*In vitro*, human	Y79, WERI-RB1	Wang et al. (2016)
miR-222	Up	VHL	RT-qPCR	Increased expression of HIF1α, promoted resistance to the chemotherapy drug VCR	*In vitro*, human, *in vivo*	ARPE-19, Y79, WERI-RB1, SO-Rb50, HXO-RB44	Li et al. (2020a)
miR-378a-3p	Down	FOXG1	RT-qPCR	Decreased viability, promoted apoptosis	*In vitro*, *in vivo*, human	Y79, SO-Rb50, SO-Rb70, HRA	Zhang and Wu, (2020)
miR-let-7a	Down	MST4	RT-qPCR	Inhibited apoptosis, increased proliferation angiogenesis, migration and invasion	*In vitro*, *in vivo*, human	Y79, WERI-RB1	Zhang et al. (2020a)
miR-657	Up	PPARA	RT-qPCR	Enhanced tumorigenesis, inhibited PPARA expression	*In vitro*, *in vivo*	−	He and Feng, (2022)
miR-98	Down	HMGA2	RT-qPCR	Inhibited proliferation, invasion and migration, suppressed EMT and Wnt/β-catenin pathway	*In vitro*, human	ARPE-19, WERI-RB1, Y79	Li et al. (2019)
miR-101	Down	EZH2	qRT-PCR	Inhibited viability, progression and EZH2 expression, promoted apoptosis	*In vitro*, human	Y79, WERI-RB1	Lei et al. (2014)
miR-26a	−	Beclin 1	RT-PCR	Promoted proliferation, suppressed apoptosis, inhibited the expression of Beclin 1	*In vitro*	Y79, WERI-RB1	Li et al. (2016b)
mir-376a	Down	caspase-3	RT-qPCR	Inhibited ATO-induced apoptosis	*In vitro*	HXO‐Rb44	Zhang et al. (2013)
miR-320a	Up	TUSC3	RT-qPCR	Increased proliferation, inhibited apoptosis	*In vitro*	ARPE-19, Y79, WERI-Rb-1	Kong et al. (2020)
miR-506-3p	Down	NEK6	qRT-PCR	Inhibited proliferation, induced apoptosis	Human, *in vitro*	WERI-RB1, HXORb44, Y79, SO-RB50, ARPE-19	Wu et al. (2018a)
miR-133a-3p	Down	CREB1	qRT-PCR	Increased apoptosis induced cell cycle arrest	*in vivo*, human, *in vitro*	WERI-RB1, Y79, SO-RB50	Li et al. (2020b)
miR-218-5p		NACC1	qRT-PCR	Inhibited proliferation by suppressing AKT/mTOR signaling	*In vitro*	WERI-RB1	Li et al. (2020c)
miR-513b-5p	Up	TRIB1	−	Inhibited proliferation, promoted apoptosis	*In vitro*	WERI-RB1, 293T	Zhang et al. (2021a)
miR-181b	Up	−	qRT-PCR	Increased proliferation	*In vitro*	HXO-RB44	Xu et al. (2011)
miR-22-3p	Down	ENO1	RT-qPCR	Inhibited proliferation, promoted apoptosis	Human, *in vitro*	Y79, WERI-RB1, SO-RB50, APRE-19	Liu et al. (2018c)
miR-34b/c			PCR	Inhibited p53 signaling, cyclin-dependent kinases and pro-apoptotic proteins	Human	-	Carvalho et al. (2017)
miR-24	Down	p14ARF	RT-PCR	Induced apoptosis and cell cycle arrest	*In vitro*	HEK-293, HEK-293T, SKOV3, HeLa	To et al. (2012)
miR-513a-5p	Down	B7-H1	RT-PCR	Promoted proliferation, suppressed apoptosis	*In vitro*	Y79	Wu et al. (2012b)
miR-138-5p	Down	PDK1	RT-qPCR	Reduced viability, invasion and migration, and induced apoptosis	*In vitro*	Y79, ARPE-19	Wang et al. (2017a)
miR-211-5p	Down	GDNF	RT-PCR	Reduced carboplatin resistance	*In vitro*	Y79	Ke et al. (2022)
miR-4516	Up	PTEN	RT-qPCR	Promoted proliferation, inhibited apoptosis	Human, *in vitro*	Y79, ARPE-19	Hao et al. (2020)
miR-130a-3p	Down	PAX6	RT-qPCR	Suppressed tumor growth and reduced VCR resistance	*In vitro*, *in vivo*	SO-Rb50, WERI-RB1, SO-Rb70, HRA, Y79	Lu et al. (2022)

^1^ Human; RB samples obtained from patients.

HDAC9 is a member of the histone deacetylase (HDAC) family, involved in transcriptional regulation, cell death, and especially in carcinogenesis and cancer progression (Marks et al., 2004; Dokmanovic and Marks, 2005; Singh et al., 2017). The level of HDAC9 was higher in RB samples, and this upregulation was correlated with tumor size, regional lymph node metastasis, and poor tumor differentiation (Zhang et al., 2016a). Patients with RB tunors with high levels of HDAC9 had shorter overall survival and progression-free survival, compared to patients who expressed low levels of HDAC9 (Zhang et al., 2016a). HDAC9 has been hypothesized to act as an oncogene in the onset of RB and its progression by affecting a range of pathophysiological pathways (Mohammad et al., 2017; Jin et al., 2018). HDAC9 has the ability to reduce EGFR expression and hence inhibit the activation of the downstream PI3K/AKT signaling pathway, which leads to the development of cancer (Watson et al., 2013).

MiR 936 expression was quantified in RB by Xu et al. Importantly, they identified the contribution of miR-936 to RB oncogenesis, and suggested some underlying molecular pathways (Sheets et al., 2020). Reverse-transcription quantitative PCR was used to assess miR-936 expression in RB tissues and cell lines. Various tests including cell counting kit 8, flow cytometry, invasion, migration, and colony formation assays, as well as subcutaneous heterotopic xenografts were used to assess cell proliferation, colony formation, apoptosis, migration, and invasion *in vitro*, and tumor growth *in vivo*. Bioinformatic analysis was used to determine the possible targets of miR-936, and RT-qPCR, luciferase reporter assays and Western blotting were used to confirm the targets. Low levels of miR-936 in RB were correlated with lymph node metastasis, differentiation, and TNM stage, in both RB tissues and cells. Exogenous miR-936 inhibited RB cell proliferation, colony formation, migration, and invasion *in vitro*, and reduced tumor growth *in vivo*, because overexpression of miR-936 increased apoptosis. It was found that the histone deacetylase 9 (HDAC9) mRNA was a direct target of miR-936. HDAC9 depletion produced effects on RB cells that were comparable to those of miR-936 overexpression. The restoration of HDAC9 expression led to a decrease in the tumor-suppressor effects of miR-936, and increased the oncogenicity of RB cells. Exogenous expression of miR-936 inhibited the activity of the PI3K/AKT pathway both *in vitro* and *in vivo*, by suppressing the expression of HDAC9 in RB cells. A poor prognosis in RB patients was correlated with downregulation of miR-936, and its overexpression decreased RB aggressiveness by reducing HDAC9 mRNA and subsequently inactivating the PI3K/AKT pathway (Xu et al., 2020a).

The polycomb repressor complex 2 catalytic core protein, also known as enhancer of zeste homolog 2 (EZH2), is a highly conserved histone methyltransferase (Kuzmichev et al., 2004). By catalyzing the trimethylation of histone H3 K27, EZH2 suppressed the expression of its target genes (Cheng et al., 2016). EZH2 has been found to be upregulated in a variety of cancers, including colorectal, prostate, and breast cancer (Varambally et al., 2002; Kleer et al., 2003; Fluge et al., 2009). According to a study by Khan et al. (Khan et al., 2015), the level of EZH2 was increased in RB specimens. It is also well known that EZH2 stimulates the growth of cancer cells. Lian et al. (Gao et al., 2021a) demonstrated that EZH2 modulated Runt-related transcription factor 3 expression in order to promote the proliferation of laryngeal cancer cells. In addition, small-molecule EZH2 inhibitors suppressed tumor growth by inducing the tumor-suppressor protein p16INK4A (Mohammad et al., 2017).

Jin et al. investigated the role of miR-101-3p in retinoblastoma and tested the hypothesis that miR-101-3p targeted histone deacetylase 9 (HDAC9) as well as EZH2 (Jin et al., 2018). Retinoblastoma specimens had been shown to have downregulated expression of miR-101-3p. MTT and flow cytometry assays were used to show that exogenous overexpression of miR-101-3p significantly inhibited viability and caused cell cycle arrest in WERI-Rb-1 and Y79 cells. *In vivo* mouse studies supported the anti-tumor activity of miR-101-3p in retinoblastoma. Furthermore, predictions from TargetScan software suggested that miR-101-3p would target the 3′-untranslated regions of both HDAC9 and EZH2 mRNAs. The results obtained from the dual luciferase reporter assay showed that miR-101-3p directly targeted EZH2 and HDAC9 to inhibit the proliferation of RB cells. The anti-proliferative effects of miR-101-3p in WERI-RB-1 and Y79 cells were abrogated by the restoration of EZH2 or HDAC9 expression. These findings underline the importance of miR-101-3p in the growth of RB tumors, and suggest a potential new therapeutic target (Zheng et al., 2020).

Sushi domain-containing 2 (SUSD2) is a type I transmembrane protein that contains an AMOP (adhesion-associated domain present in MUC4 and other proteins) domain. The AMOP domain is also found in somatomedin B and von Willebrand factor type D, which are proteins involved in cell-cell and cell-matrix adhesion (Watson et al., 2013). Previous studies have shown that SUSD2 can function as a tumor suppressor in a wide range of cancers. It was found that SUSD2 could play both a negative and a positive role in high-grade serous ovarian cancer. SUSD2 was negatively associated with metastasis, but was positively associated with a longer survival time (Sheets et al., 2020). Additionally, lung adenocarcinoma patients with low SUSD2 expression levels have shorter survival, so SUSD2 may function as an independent prognostic indicator (Guo et al., 2020). In lung cancer and RCC, lower SUSD2 expression also acted as a tumor suppressor (Cheng et al., 2016).

Liu et al. performed both *in vitro* and *in vivo* studies on the development of retinoblastoma and the involvement of angiogenesis. In these studies, they investigated the putative function and mechanism of the miR-141-3p/SUSD2 axis (Liu and Wen, 2022). They used the Gene Expression Omnibus (GEO) datasets, to identify the expression levels of SUSD2 and miR-141-3p in retinoblastoma samples compared to healthy controls. The biological role and molecular mechanism of the miR-141-3p/SUSD2 axis in RB development and progression were investigated using bioinformatics analysis, a dual-luciferase reporter assay, and loss, gain, and rescue of function assays. Their findings demonstrated that RB tissues and cells expressed significantly lower levels of SUSD2. *In vitro*, an increased level of SUSD2 inhibited RB cell viability, promoted apoptosis, and prevented primary human umbilical vein endothelial cells (HUVECs) from forming tubes. The results of the dual-luciferase reporter assay and bioinformatics analysis demonstrated that miR-141-3p directly regulated SUSD2. MiR-141-3p overexpression accelerated angiogenesis, whereas miR-141-3p suppression inhibited RB development. Both *in vivo* and *in vitro* overexpression of SUSD2 partially reversed these effects. They concluded that SUSD2 acts as a RB tumor suppressor, and the miR-141-3p/SUSD2 axis could regulate both retinoblastoma development and angiogenesis, making it a new target for RB treatment (Liu and Wen, 2022).

## 3 Long non-coding RNAs and retinoblastoma

Long non-coding RNAs (lncRNAs) are more than 200 nucleotides in length (Silva et al., 2019; Wang et al., 2021a). LncRNAs are involved in many pathways that affect tumor biology, and have major roles in controlling oncogenes as well as tumor suppressor genes (Huang et al., 2017). For instance, LINC00504 was increased in breast cancer, where it promotes tumor cell proliferation and migration (Hou et al., 2021). By acting as a competing endogenous RNA (ceRNA) or sponge, lncRNAs can affect gene transcription. CeRNAs can specifically sponge their matching miRNA to control expression of the mRNAs of the relevant target genes (Chen et al., 2021a; Wang et al., 2021b). These regulated target genes can play a role in several signaling pathways, almost all of which are strongly linked to tumors (Gao et al., 2021c). It has been confirmed that certain lncRNAs are involved in the progression of human tumors (Tsai et al., 2011; Tang et al., 2013), including retinoblastoma (Wang et al., 2018a). Numerous human cancers, such as hepatocellular carcinoma (HCC) (Li and Zheng, 2017), colorectal cancer (CRC) (Yan et al., 2020), and prostate cancer have been shown to be affected by the oncogenic roles of the actin-binding LIM and SH3 domain protein 1 (LASP1) (Wang et al., 2017c). In an investigation into oral cancer, it was revealed that silencing of LASP1 resulted in cell cycle arrest in G2 phase (Shimizu et al., 2013). Additionally, in clear cell renal cell carcinoma, LASP1 silencing inhibited cell migration (Yang et al., 2014). MiRNAs have been hypothesized to regulate LASP1 in the initiation and progression of various cancers (INVALID CITATIONa; Hu et al., 2017a). The development of oral squamous cell carcinoma was affected by LASP1, a miR-342-3p downstream target, according to a study by Song et al. (Nordlund and Reichard, 2006). Yang et al. (Yang et al., 2019b) also showed that lncRNA SNHG16 control led LASP1 to promote RB cell invasion and migration.

Xu et al. examined the regulatory mechanism and potential role of the lncRNA, myocardial infarction-associated transcript (MIAT) in RB progression (Xu et al., 2021b). The expression of MIAT, miR-665, LASP1, and other proteins were measured using RT-qPCR in RB cells or patient tissues. The dual-luciferase reporter assay was used to confirm the interactions between miR-665 and MIAT/LASP1. Western blotting, MTT and transwell assays were used *in vitro* to investigate the effects of the MIAT/miR-665/LASP1 axis on RB development. Their findings showed that MIAT specifically targeted miR-665. High MIAT expression was found in cell lines and RB tissues, while miR-665 was downregulated in RB tissues. In addition, either miR-665 overexpression or MIAT downregulation led to reduced proliferation, migration, and invasion of RB cells, HXO-RB44 and Y79. LASP1 was also suggested to be a target gene for miR-665. Both downregulation of miR-665 and upregulation of LASP1 reversed the suppressive effects of MIAT knockdown on the proliferation, migration, and invasion of Y79 cells. Additionally, MIAT silencing prevented RB proliferation by regulating the miR-665/LASP1 axis. They suggested that MIAT might be a promising target for RB treatment (Grossi et al., 2015).

Brain-derived neurotrophic factor (BDNF) is involved in the survival, differentiation, growth, and development of neurons (INVALID CITATIONa; McAllister, 2001). In cancer biology, the expression of BDNF was found to be elevated in gliomas, breast cancer, colorectal cancer, gastric cancer, bladder cancer, and other human tumors (Colucci-D’Amato et al., 2020). In colon cancer, BDNF promoted cancer progression by increasing the expression of HO-1 and transcription of VEGF, as well as the activation of the MAPK signaling pathway (Huang et al., 2015). BDNF promoted cancer development by activating tropomyosin receptor kinase B (TrkB) in triple-negative breast cancer (Tsai et al., 2017). High levels of BDNF increased the proliferation and invasion of RB cells by triggering the PI3K/AKT signaling pathway (INVALID CITATIONa).

Xu et al. investigated the function of the lncRNA XIST, and its underlying mechanism in RB (Xu et al., 2021c). In this investigation, RT-qPCR and Western blotting were used to measure the levels of XIST, miR-191-5p, BDNF mRNA, and BDNF protein in RB tissues and cell lines. In order to carry out gain-of-function and loss-of-function experiments, they transfected pcDNA3.1-XIST, XIST siRNA, and miR-191-5p mimics and inhibitors into SO-Rb50 and Y79 cells. Several methods including CCK-8, transwell, and terminal deoxynucleotide transferase UTP nick-end labeling (TUNEL) experiments were employed to measure RB cell proliferation, invasion, migration, and apoptosis. The use of luciferase reporter assays, RT-qPCR, Western blotting, and bioinformatics analysis, allowed the regulatory relationships between BDNF, XIST, and miR-191-5p to be elucidated. They found that XIST expression was significantly increased in RB tissues and cells. High expression of XIST promoted proliferation, invasion, migration, and inhibited apoptosis in RB cells, but miR-191-5p showed the opposite effects. Furthermore, miR-191-5p inhibited the expression of BDNF at both mRNA and protein levels. XIST indirectly increased the expression of BDNF by acting as a ceRNA and inhibiting miR-191-5p expression. They concluded that the expression level of XIST was increased in RB tissues, and XIST could control proliferation, invasion, migration, and apoptosis through regulating the miR-191-5p/BDNF axis (Xu et al., 2021c).

The hypoxia-inducible factor-1α (HIF-1α) gene is located on chromosome 14q23.2. HIF-1α is a crucial subunit of hypoxia-inducible factor-1 (HIF-1). Numerous studies have confirmed the oncogenic role of HIF-1α. For example, an interaction between HIF-1α and LOXL2 (lysyl oxidase like 2) in hepatocellular carcinoma promoted cancer development, and increased angiogenesis and the EMT (Wang et al., 2017b). Elevated expression of HIF-1α in thyroid follicular carcinoma was associated with distant metastasis (Klaus et al., 2018). HIF-1α also acts as an oncoprotein in RB, and its knockdown led to increased expression of pro-apoptotic proteins, including Bax, caspase-9, and caspase-3, thus triggering apoptosis in cancer cells (Gao et al., 2014). In addition, HIF-1α increased invasion of RB cells by inducing MMP-9 expression. (Li and Zheng, 2017). Besides, it has been shown that activation of HIF-1α can increase resistance to various cancer therapies (Liang et al., 2013; Wang et al., 2017c). Investigations by Dong et al. (Wang et al., 2017c) and Greco and Scott (Greco and Scott, 2007) both showed that HIF-1α plays a role in the increased survival of tumor cells in response to chemotherapy and/or radiotherapy.

Yan et al. (Yan et al., 2022) investigated the role of the lncRNA MIR17HG (miR-17-92a-1 cluster host gene) and its interaction with miR-155-5p and HIF-1α pathway in RB development. qRT-PCR showed that up-regulation of MIR17HG was negatively associated with miR-155-5p expression. CCK-8 and transwell assays showed that overexpression of MIR17HG increased proliferation, migration, and invasion of RB cells. MiR-155-5p was a MIR17HG target which could inhibit growth, migration, and invasion of RB cells. Furthermore, MIR17HG enhanced the gene and protein expression of HIF-1α in RB cells. Taken together, this study suggested the oncogenic effects of MIR17HG in RB mediated through the miR-155-5p/HIF-1α axis (Yan et al., 2022).

Ribonucleotide reductase M2 subunit (RRM2) catalyzes the rate-limiting step of DNA synthesis and repair, and has important effects on multiple cell processes, including proliferation, migration, invasion, and senescence (Nordlund and Reichard, 2006). In addition, upregulation of RRM2 acted as a cancer driver in different malignancies (Grossi et al., 2015; Grolmusz et al., 2016). In adrenocortical cancer, RRM2 was strongly associated with Ki67 expression (Grolmusz et al., 2016). In pancreatic cancer, RRM2 increased the expression of Bcl-2, an anti-apoptotic protein, and decreased cleaved caspase-3 (Xia et al., 2017). In RB, enhanced expression of RRM2 affected RB cell cycle progression (Nie et al., 2021).

To investigate the underlying mechanism of the lncRNA HOTAIR in RB development, Fu et al., (Fu et al., 2022), showed that HOTAIR was the upregulated in RB cells (ORB50, Y79, HXO-RB44, and WERI-RB) in comparison with normal retinal cells (ARPE-19 and RPE-1). HXO-RB44 and Y79 cells had relatively higher expression of this marker. In the next step, delivery of sh-HOTAIR into RB cells (Y79 and HXO-RB44) arrested the cell-cycle, inhibited proliferation, and increased apoptosis, as detected by CCK-8 assay and flow cytometry. Dual-luciferase assay reveled that HOTAIR acted as a ceRNA of miR-20b-5p and could also increase the expression of RRM2. Besides, functional rescue experiments showed that downregulation of miR-20b-5p or upregulation of RRM2 could promote proliferation and the RB cell-cycle, inhibit apoptosis, and reverse the effects of sh-HOTAIR on inhibition of RB cells. Treatment of a xenograft tumor model with sh-HOTAIR not only decreased tumor development and the number of Ki67 + cells, but also inactivated the PI3K/AKT axis. LncRNA HOTAIR competitively bound to miR-20b-5p, and therefore upregulated RRM2 and activated the PI3K/AKT pathway to enhance RB cell proliferation and suppress apoptosis (Fu et al., 2022).

High-mobility group box 1protein (HMGB1) is a nuclear protein with cytokine-like activity, which was secreted by neurons following ethanol exposure (Crews et al., 2013; Zou and Crews, 2014). HMGB1 can activate immune responses to TLR7 agonists (Yanai et al., 2009), and functions as a chaperone for cytokines or DNA, and improves their interactions with their normal receptors (Boonyaratanakornkit et al., 1998; Sha et al., 2008; Bianchi, 2009). HMGB1 is released inside microvesicles (MVs) from macrophages and its expression and active secretion in the brain are increased in response to ethanol (Ardoin and Pisetsky, 2008; Zou and Crews, 2014). The expression of the HMGB1 gene located chromosomal region 8q22, is increased in RB, and is correlated with poorly differentiated tumor and invasion of the optic nerve (Singh et al., 2015). HMGB1 was found to stimulate RB tumorigenesis and potentiate its malignant properties (Liu et al., 2014; Wang et al., 2017d; Chai et al., 2018; Liu et al., 2018d).

Zhang et al., (Zhang et al., 2020b), examined the expression of LINC00205 in RB specimens to uncover its exact role in RB tumorigenesis. They found that high expression of LINC00205 in RB cells and tissues was associated with unfavorable clinicopathological properties and shorter overall survival times in RB patients. LINC00205 knockdown *in vitro* inhibited proliferation and stimulated apoptosis in RB cells, while it slowed RB tumor development *in vivo*. Mechanistically, LINC00205 increased the expression of the miR-665 target mRNA HMGB1, because it acted as a ceRNA to sponge miR-665 in RB cells. If the miR-665-HMGB1 pathway was activated, it weakened the effects of LINC00205 depletion in RB cells. Therefore, the LINC00205/miR-665/HMGB1 axis could be a useful target for RB prognosis, diagnosis, and even therapy (Zhang et al., 2020b).

E2F transcription factor 3 (E2F3) is a member of the E2F family of transcription factors which are classified into three main groups; atypical inhibitors (E2F7 and E2F8), canonical inhibitors (E2F3b-E2F6), and activators (E2F1, E2F2, and E2F3a) of gene transcription (Kent and Leone, 2019). The atypical and the canonical inhibitors prevent gene transcription and act as tumor suppressors (Kent and Leone, 2019). There are lines of evidence showing that miRNAs, such as miR-145-5p can regulate E2F3 (Zehavi et al., 2015; Hu et al., 2017b). E2F3 is involved in various human tumors as a tumor promoter (Wang et al., 2019c). E2F3 was found to be upregulated in RB (Madhavan et al., 2009; Zhao et al., 2020a), while its downregulation was associated with the inhibition of RB cell proliferation (Zhao et al., 2020a).

Zhang et al., (Zhang et al., 2020c), investigated the effects of the lncRNA CASC9 on RB cells malignant properties, such as proliferation, invasion, EMT, and apoptosis. CASC9 overexpression noticeably promoted the malignant phenotype of RB cells. By contrast, silencing of CASC9 suppressed the malignant potential of RB cells and increased apoptosis. RNA immunoprecipitation and dual-luciferase reporter assays demonstrated that CASC9 could regulate E2F3 *via* sponging miR-145-5p. In more detail, the effects of CASC9 knockdown could be reversed in part by inhibition of miR-145-5p or overexpression of E2F3. Moreover, the overexpression of miR-145-5p could further promote properties already stimulated by CASC9 silencing. All of which suggest that CASC9 could be a therapeutic target to control RB (Table 2) (Zhang et al., 2020c).

**TABLE 2:** The role of lncRNAs in retinoblastoma.

LncRNA	Expression status	Target	Detection method	Effect	Model	Cell line	Reference
KCNQ1OT1	Up	miR-124	RT-qPCR	Increased proliferation, migration, and cell cycle, reduced apoptosis	Human^1^, *in vitro*, *in vivo*	Y79, WERI-RB1, hTERT RPE-1	Zhang et al. (2021b)
	Up	miR-134	qRT-PCR	Promoted proliferation, migration and invasion	Human, *in vitro*, *in vivo*	WERI-RB1, Y79, ARPE-19	Wang et al. (2021c)
	Up	miR-153-3p	qRT-PCR	Promoted proliferation, migration and invasion	Human, *in vitro*	SO-Rb50, HXO-RB44	Wang et al. (2020a)
XIST	Up	miR-140-5p	qRT-PCR	Promoted proliferation and invasion	Human, *in vitro*	ARPE-19, Y79, WERI-RB1, SO-Rb50, HXO-RB44	Wang et al. (2020b)
	Up	miR-142-5p	qRT-PCR	Promoted proliferation and epithelial-mesenchymal transition	Human, *in vitro*	WERI-RB1, HXO-RB44, SO-RB50, ARPE-19, Y79	Xu and Tian, (2020)
	Up	miR-191-5p	qRT-PCR	Promoted proliferation, migration, invasion, and reduced apoptosis	Human, *in vitro*	HXO-RB44, WERI-RB-1, SO-RB50, ARPE-19, Y79	Xu et al. (2021d)
	Up	miR-204-5p	qRT-PCR	Enhanced proliferation and autophagy, induced vincristine resistance	Human, *in vitro*, *in vivo*	WERI-RB1, Y79, ARPE-19	Yao et al. (2020)
	Up	miR-200a-3p	qRT-PCR	Induced EMT, proliferation and invasion, inhibited apoptosis	Human, *in vitro*	Y79, ARPE-19	Zhao et al. (2020b)
	Up	miR-361-3p	qRT-PCR	Promoted proliferation, migration, invasion and autophagy, suppressed apoptosis	*In vitro*, human, *in vivo*	Y79, WERI-RB1, ARPE-19	Yang et al. (2020c)
	Up	miR-101	RT-PCR	Promoted proliferation, migration, invasion and EMT, suppressed apoptosis, reduced caspase-3 activity	*In vitro*, human	SO-RB50, Y79, WERI-RB1, ARPE-19	Cheng et al. (2019)
FEZF1-AS1	Up	miR-363-3p	qPCR	Promoted viability and proliferation, reduced apoptosis	Human, *in vitro*	WERI-RB1, Y79, ARPE-19	Liu et al. (2021b)
	Up	miR-1236-3p	RT-qPCR	Promoted EMT, migration and invasion	*In vitro*	ARPE-19, Y79, SO-Rb50, WERI-RB1, RBL-13	Zhang et al. (2020d)
UCA1	Up	miR-513a-5p	qRT-PCR	Increased proliferation and multidrug resistance	Human, *in vitro*	SO-RB50, HEK-293T	Yang et al. (2020d)
LINC00858	Up	miR-3182	RT-qPCR	Promoted proliferation, migration and invasion	Human, *in vitro*	SO-RB50, Y79, HXO-RB44, WERI-RB1, ARPE-19	Wang et al. (2020c)
LINC00152	Up	MiR-613	RT-qPCR	Increased aggressiveness; Enhanced carboplatin and adriamycin Resistance	Human, *in vitro*, *in vivo*	Y79, WERI-RB1, ARPE-19, RBL-13, SO-RB50	Wang et al. (2020d)
LincRNA-ROR	Up	miR-32-5p	qRT-PCR	Correlated with optic nerve invasion, nodal or distant metastasis, and recurrence	Human, *in vitro*, *in vivo*	hTERT RPE-1, WERI-RB1, Y79	Gao et al. (2021d)
MIAT	Up	miR-665	RT-qPCR	Increased proliferation, migration and invasion	Human, *in vitro*	ARPE-19, HXO-RB44, WERI-RB1, SO-RB50, Y79	Xu et al. (2021b)
CCAT1	Up	miR-218-5p	Q-PCR	Promoted EMT, migration and invasion	Human, *in vitro*	SO-RB50, Y79, WERI-RB1, ARPE-19	Meng et al. (2021)
	Up	miR-218-5p	qRT-PCR	Promoted proliferation, migration and invasion, reduced apoptosis	*In vitro*	SO-RB50, Y79, WERI-RB1	Zhang et al. (2017b)
LINC00202	Up	miR-204-5p	qRT-PCR	Promoted tumor progression by regulating proliferation, apoptosis and aerobic glycolysis	Human, *in vitro*, *in vivo*	Y79, HXO-RB44	Wu et al. (2020)
	Up	miR-3619-5p	qRT-PCR	Promoted proliferation, migration and invasion	*In vitro*, human	Y79, WERI-RB1, SORB50, hTERT-RPE1, ARPE-19, HXO-RB44	Yan et al. (2019)
SNHG20	Up	miR-335-5p	RT-qPCR	Promoted proliferation, migration and invasion	Human, *in vitro*	WERI-RB1, SO-RB50, ARPE-19, Y79	Song and Zhang, (2021)
SND1-IT1	Up	miR-132-3p	RT-qPCR	Promoted proliferation, invasion and migration	Human, *in vitro*, *in vivo*	WERI-RB1, SO-RB50, ARPE-19, Y79	Yin et al. (2021)
TMPO-AS1	Up	miR-199a-5p	qRT-PCR	Promoted proliferation, migration and invasion	Human, *in vitro*	HXO-RB44, SO-Rb50	Peng et al. (2020)
DANCR	Up	miR-34c, miR-613	RT-PCR	Promoted proliferation, migration, invasion, and EMT, increased N-cadherin, vimentin)	Human, *in vitro*, *in vivo*	WERI-RB1, SO-RB50, Y79, HXO-RB44	Wang et al. (2018b)
MBLN1-AS1	Down	miR-338-5p	RT-qPCR	Inhibited Wnt/β-catenin signaling pathway, inhibited proliferation and migration	*In vitro*, *in vivo*	Y79, WERI-RB1, ARPE-19, SO-RB	Xu et al. (2021e)
HOTTIP	Up	miR-101-3p	qRT-PCR	Promoted proliferation, inhibited apoptosis	Human, *in vitro*, *in vivo*	Y79, HXO-RB-44, ARPE-19, SO-RB50	Yuan et al. (2021)
HOTAIR	Up	miR-20b-5p	qRT-PCR	Promoted proliferation, inhibited apoptosis	*In vivo*, *in vitro*	ARPE-19, RPE-1, SORB50, Y79, HXO-RB44, WERI-RB1	Fu et al. (2022)
ILF3-AS1	Up	miR-132-3p	RT-qPCR	Enhanced proliferation and invasion	Human, *in vitro*, *in vivo*	ARPE-19, Y79, HXO-RB44, SO–Rb50, Rb1	Han et al. (2020)
MALAT1	Up	miR-124	qRT-PCR	Induced proliferation, migration and invasion, inhibited apoptosis	*In vitro*	Y79, ARPE-19	Liu et al. (2018e)
	Up	miR-20b-5p	RT-qPCR	Promoted proliferation, suppressed apoptosis	*In vitro*, human, *in vivo*	SO-RB50, ARPE-19, HXO-RB44, WERI-RB1, Y79	Wang et al. (2020e)
LINC00205	Up	microRNA-665	RT-qPCR	Promoted proliferation, invasion and migration, reduced apoptosis, promoted tumor growth	Human, *in vitro*, *in vivo*	Y79, SO-RB50, WERI-RB1, ARPE-19	Zhang et al. (2020b)
PVT1	Up	miR-488-3p	qRT-PCR	Induced proliferation, migration, invasion, and cell cycle progression, inhibited apoptosis	Human, *in vitro*, *in vivo*	SO-RB50, WERI-RB1, Y79, HXO-RB44, ARPE-19	Wu et al. (2019b)
H19	Down	miR-17–92	qRT-PCR	Inhibited proliferation, induced cell cycle arrest and apoptosis	Human, *in vitro*	ARPE-19, Y79, WERI-RB1, SO-RB50	Zhang et al. (2018d)
MIR17HG	Up	miR-155-5p	qRT-PCR	Promoted proliferation, migration, and invasion	Human, *in vitro*	HXO-RB44, SO-Rb50, Y79, WERI-RB1, ARPE-19	Yan et al. (2022)
PROX1-AS1	Up	miR-519d-3p	−	Induced drug resistance, proliferation, migration and invasion, inhibited apoptosis	Human, *in vitro*	−	Chen et al. (2021b)
MIR7-3HG	Up	miR-27a-3p	qRT-PCR	Promoted proliferation, suppressed apoptosis	*In vitro*, human	Y79, WERI-RB1, ARPE-19	Ding et al. (2020)
TP53TG1	Up	miR-33b	qPCR	Promoted proliferation, migration and invasion	*In vitro*, human, *in vivo*	SO-RB50, WERI-RB1, Y79, RBL-13, ARPE-19	Wang et al. (2021d)
FOXD2-AS1	Up	miR-31	qRT-PCR	Inhibited proliferation and migration	*In vitro*, human	SO-RB50, Y79, WERI-RB1, ARPE-19	Liang et al. (2022a)
CASC9	Up	miR-145-5p	qRT-PCR	Promoted proliferation, invasion and EMT, suppressed apoptosis	*In vitro*, human	Y79, WERI-RB1, ARPE-19	Zhang et al. (2020c)
RHPN1-AS1	Up	miR-3133	qRT-PCR	Promoted viability, suppressed apoptosis	*In vitro*	Y79, WERI-RB1, ARPE-19	Li et al. (2020d)
TP73-AS1	Up	miR-139-3p	RT-qPCR	Promoted proliferation	*In vitro*, human	WERI-RB1, Y79	Xia et al. (2019b)
ANRIL	Up	miR-328	qRT-PCR	Increased resistance to DDP, proliferation, upregulated ABCG2 and MDR1, inhibited apoptosis	*In vitro*	HXO-RB44, Y79	Yin et al. (2020)
ZFPM2-AS1	Up	miR-515	qRT-PCR	Promoted proliferation and metastasis, activated Wnt/β-catenin signaling	*In vitro*, *in vivo*, human	WERI-RB1, SO-RB50, Y79, ARPE-19	Lyv et al. (2020)
	Up	miR-511-3p	qRT-PCR	Promoted viability and migration	*In vitro*, human	SO-RB50, WERI-RB1, HXO-RB44, Y79, ARPE-19	Ni et al. (2022)
HCP5	Up	miR-3619-5p	RT-qPCR	Promoted proliferation, migration and invasion	*In vitro*, *in vivo*, human	Y79, WERI-RB1, HXO-RB44, ARPE-19, SO-RB50	Zhu and Hao, (2021)
LINC00324	Up	miR-769-5p	RT-qPCR	Promoted proliferation, colony formation, migration and invasion, suppressed apoptosis	*In vitro*, *in vivo*, human	Y79, SO-RB50, WERI-RB1, ARPE-19	Dong et al. (2020)
FTX	Up	miR-320a	qRT-PCR	Exacerbated aggressive phenotype, promoted proliferation, migration and invasion, promoted RB tumor growth	*In vitro*, *in vivo*, human	Y79, SO-RB50, WERI-RB1, ARPE-19	Wang et al. (2021e)
LINC00488	Up	miR-30a-5p	RT-qPCR	Increased tumorigenicity and malignant phenotype	Human, *In vivo*, *In vitro*	SO-RB-50, WERI-RB1, ARPE-19, Y79	Cui et al. (2022)
NEAT1	Up	miR-204	-RT-PCR	Increased proliferation and migration, decreased apoptosis	*In vitro*, *in vivo*, human	Y79, WERI-RB1, SO‐RB50, ARPE‐19	Zhong et al. (2019)
TUG1	Up	−	qPCR	Promoted proliferation, migration, invasion, and induced EMT	Human, *in vitro*	WERI-RB1, Y79, SO-RB50, RBL-13, ARPE-19	Wang et al. (2022)

^1^ Human; RB samples obtained from patients.

## 4 Circular RNAs and retinoblastoma

CircRNAs are a subclass of endogenous ncRNAs, which were first misinterpreted as by-products of splicing errors (Sanger et al., 1976). Recently however, it has been shown that circRNAs are derived from intronic or exonic sequences by back-splicing, and then form a stable covalently closed circular loop without any 5′ end caps or 3′ end poly(A) tails (Wang et al., 2017e). The majority of circRNAs are evolutionarily conserved, widespread, abundant, and stable, and they show tissue or developmental specificity in eukaryotes (Bahn et al., 2015; Wang et al., 2017f). In contrast to linear RNAs, circRNAs are not sensitive to exonuclease digestion, and can resist the degradation suffered by linear RNA due to their distinctive single-stranded closed circlular loop. Their presence in different samples such as serum, tissues, and urine makes them useful as biomarkers for various human cancers, as well as age-related disorders (Memczak et al., 2013; Fang et al., 2019). It is accepted that circRNAs are involved in several human diseases such as cancer, because of their ability to alter the malignant properties of cancer cells and their response to chemotherapy agents (Li et al., 2015). Therefore, circRNAs could be novel therapeutic targets to manage various tumors (Chen, 2016; Shao et al., 2018; Drula et al., 2020; Li et al., 2020e; Luo et al., 2020) including pancreatic cancer (Sharma et al., 2021).

WNT3A is an important element in the Wnt/β-catenin pathway, which contributes to proliferation, differentiation, and carcinogenesis (Yun et al., 2005; He et al., 2015). WNT3A expression levels were associated with cancer cell proliferation and drug-resistance *via* Wnt/β-catenin signaling pathway (INVALID CITATIONb). The overexpression of miR-15a-5p could target WNT3A mRNA leading to inhibition of proliferation and stemness of human endometrial adenocarcinoma (HEC-1-A) cells (Zhang et al., 2007). Moreover, miR-485 could inhibit WNT3A in RB cells and further inhibit Wnt/β-catenin signaling in these cells (INVALID CITATIONb).

Wang et al. (Wang et al., 2020f) reported that circDHDDS was upregulated in RB tissues and cells (Y-79, RPCs, and WERI-RB1) when compared to retinal pigment epithelial cells and normal retinal tissues. Colony formation, transwell, and flow cytometry assays showed that knockdown of circDHDDS inhibited the malignant potential of RB cells, and caused cell cycle arrest. Similar results were found in a RB xenograft model following silencing of circDHDDS. To elucidate the molecular mechanism by which circDHDDS promoted RB progression, bioinformatics databases and a dual-luciferase reporter assay revealed that circDHDDS could sponge miR-361-3p which in turn targeted WNT3A. Consequently, miR-361-3p overexpression inhibited WNT3A expression resulting in inhibition of RB progression. Thus, the circDHDDS/miR-361-3p/WNT3A pathway promoted RB progression and increased proliferation, migration, invasion, and the cell cycle in RB cells (Wang et al., 2020f).

It was found that Syntaxin 17 (STX17) could bind to vesicle-associated membrane protein 8 (VAMP8) and synaptosome-associated protein 29 (SNAP29) to promote the fusion of autophagosomes with lysosomes by modulating the autophagosome membrane (Itakura et al., 2012; Uematsu et al., 2017). STX17 is a localized endoplasmic reticulum membrane protein, which affects cell survival *via* interactions with Fis1, ATG14L, and BAP31 to form a functional complex (Wang et al., 2008; Hamasaki et al., 2013; Machihara and Namba, 2019). Huang et al. investigated the effect of STX1 on RB cell autophagy *via* the lncRNA MALAT1/miR-124 axis (Huang et al., 2018b). Upregulation of STX17 in RB cells increased autophagy (Huang et al., 2018b).

Liu et al. reported the increased expression of circ_0000034 and STX17 as well as the reduced expression of miR-361-3p in RB cells and tissues. Silencing of circ_0000034 inhibited proliferation, migration, invasion, autophagy, and tumor growth, and induced death in RB cells. The use of dual-luciferase reporter assays and RNA immunoprecipitation demonstrated an interaction between circ_0000034 and miR-361-3p, as well as an interaction between miR-361-3p and STX17. Circ_0000034 overexpression and miR-361-3p depletion both resulted in increased expression of STX17, and promoted the progression of RB. On the other hand, circ_0000034 knockdown inhibited RB progression *via* modulating the miR-361-3p/STX17 axis (Liu et al., 2020c).

A Disintegrin and Metalloproteinase 19 (ADAM19) is a member of the ADAM family, which is highly expressed in different tumors (Wildeboer et al., 2006; Chan et al., 2008; Zhang et al., 2015a; Zhang et al., 2019), including NSCLC cells (Wang et al., 2019d). ADAM19 is a transmembrane protein which contributes to tumor development, such as glioma and colorectal cancer (Qi et al., 2009). ADAM19 was found to affect the proliferation and invasion of RB cells (Sun et al., 2015). Notably, miR-145 overexpression could target ADAM19 mRNA and reverse its effects on RB development (Sun et al., 2015).

Jiang et al. (Jiang et al., 2021a) used qRT-PCR to show that circ_0000034 expression was increased in RB tissues and cells. Silencing of circ_0000034 not only inhibited proliferation, migration, invasion, and EMT of RB cells as shown by CCK-8 and transwell assays, but also stimulated apoptosis as shown by flow cytometry. RB growth in an animal model was inhibited by silencing of circ_0000034. Using dual-luciferase reporter, RIP, and RNA pull-down assays, it was shown that circ_0000034 could sponge miR-361-3p, and thereby reverse its effects on ADAM19 in RB cells. Moreover, miR-361-3p suppression abolished the effects of silencing circ_0000034 on the malignant properties of RB cells. Overexpression of ADAM19 reversed the effects of the miR-361-3p mimic on the survival, migration, invasion, apoptosis, and EMT of RB cells. They concluded that circ_0000034 promoted RB tumorigenesis *via* the miR-361-3p/ADAM19 axis, which could therefore be a target for RB therapy (Jiang et al., 2021a).

It has been shown that Rho-associated protein kinase 1 (ROCK1) affects tumor development by regulating cellular processes, including proliferation, migration, invasion, apoptosis, and the EMT (Abe et al., 2014; Zhang et al., 2015b; Xiang et al., 2015; Leonel et al., 2017). Wang et al. reported that ROCK1 knockdown could inhibit the adhesion and invasion of RB cells (Wang et al., 2014). ROCK1 could also increase proliferation and metastasis of RB cells, and inhibit apoptosis (Wu et al., 2018b; Wang et al., 2019a). In terms of function, ROCK1 suppression could decrease the oncogenic potential of RB cells (Wang et al., 2014).

Huang et al. (Huang et al., 2021) investigated the mechanism of circ-E2F3 in RB progression. They used qRT-PCR analysis to show that circ-E2F3 was overexpressed in RB tissues and cells. Circ-E2F3 silencing inhibited proliferation, migration, and invasion of RB cells as shown by MTT, transwell, colony formation, and scratch wound healing assays. In addition, circ-E2F3 knockdown increased apoptosis in RB cells as shown by flow cytometry. Depletion of circ-E2F3 in xenograft models of RB decreased tumor growth. A dual-luciferase reporter assay showed that circ-E2F3 could sponge miR-204-5p, which in turn targeted ROCK1 mRNA. Inhibition of miR-204-5p could promote the stimulatory effects of circ-E2F3 on RB progression. Taken together, circ-E2F3 could promote RB progression *via* the miR-204-5p/ROCK1 axis (Huang et al., 2021).

The L-type amino acid transporter (LAT1) can regulate cancer cell functions, such as apoptosis, proliferation, and drug-resistance by activation of the downstream AKT/mTOR pathway (Rosilio et al., 2015; Grzes et al., 2017). He et al. demonstrated that upregulation of miR-184 reduced the expression of solute carrier family 7 member 5 (SLC7A5, another term for LAT1) and reduced the proliferation, migration and invasion of RB cells (He et al., 2019). SLC7A5 expression levels tended to be higher in RB tissue samples (He et al., 2019).

Zheng et al., examined the effects and functional mechanism of the circRNA ER membrane protein complex subunit 9 (circ-FAM158A) in RB cells (Zheng et al., 2021). They first analyzed the expression levels of miR-138-5p, circ-FAM158A and SLC7A5 in RB samples by qRT-PCR. They found that circ-FAM158A and SLC7A5 were both upregulated, while miR-138-5p was downregulated in RB tissues. Knockdown of circ-FAM158A reduced the oncogenic properties of RB cells as shown by by CCK-8, colony formation, and transwell assays. Depletion of circ-FAM158A also stimulated apoptosis in RB cells as shown by by flow cytometry. A mouse xenograft model was used to confirm the role of circ-FAM158A in RB progression. By using StarBase and a dual-luciferase reporter assay, the molecular interplay between circ-FAM158A, miR-138-5p, and SLC7A5 was confirmed. They showed that circ-FAM158A could not only sponge miR-138-5p, but also upregulate its target SLC7A5. Functionally, miR-138-5p inhibition could reverse the anti-cancer effects of the silencing of circ-FAM158A on RB progression. Also, SLC7A5 overexpression blocked the anti-cancer effects of miR-138-5p in RB cells. Therefore, the anti-tumor effects of circ-FAM158A knockdown in RB cells occurred *via* the miR-138-5p/SLC7A5 axis (Zheng et al., 2021).

The SMAD family member 2 (SMAD2) gene is located on 18q21.1, and is a crucial signal transducer of the TGF-β pathway. Inhibition of SMAD2 resulted in the prevention of EMT progress, and reduced the proliferation and invasion stimulated by the TGF-β signaling pathway (Isselbacher et al., 2016; Tang et al., 2018). SMAD2 upregulation could promote the growth and metastasis of RB cells (Asnaghi et al., 2019). By contrast, SMAD2 inhibition reduced RB cell proliferation and invasion (Asnaghi et al., 2019).

To explore the function and mechanism of circ_0000527 in RB, Liang et al. (Liang et al., 2022b), measured the expression levels of circ_0000527, miR-1236-3p and SMAD2 using qRT-PCR. Their findings showed that upregulation of circ_0000527 in RB tissue samples was linked to clinicopathological features such as advanced TNM stage, and choroidal or optic nerve invasion. Also, circ_0000527 knockdown had anti-tumor effects in RB cells, with reduced proliferation, migration, invasion, and angiogenesis as shown by CCK-8, EdU, colony formation, scratch wound healing, transwell, and endothelial cell tube formation assays, respectively. In addition, flow cytometry provided evidence that silencing of circ_0000527 increased apoptosis in RB cells. Dual-luciferase reporter as well as RIP assays showed that circ_0000527 could sponge miR-1236-3p, which targeted SMAD2. Inhibition of miR-1236-3p reversed the inhibitory effects of circ_0000527 knockdown on RB malignant properties. Furthermore, overexpression of miR-1236-3p abrogated the effects of SMAD2 on RB progression. Moreover, *in vivo* experiments showed that circ_0000527 knockdown inhibited tumor formation. This study showed the role of the circ_0000527/miR-1236-3p/SMAD2 axis in RB progression (Table 3) (Liang et al., 2022b).

**TABLE 3:** The role of circRNAs in retinoblastoma.

CircRNA	Expression status	Target	Detection method	Effect	Model	Cell line	Reference
circTET1	Down	miR-492, miR-494-3p	qRT-PCR	Inhibited proliferation, migration and invasion, promoted apoptosis and cell cycle arrest	Human^1^, *in vitro*, *in vivo*	Y79, WERI-RB1, ARPE19	Fu et al. (2021)
circDHDDS	Up	miR-361-3p	RT-qPCR	Promoted proliferation, migration, and invasion, and reduced cell cycle arrest	Human, *in vitro*, *in vivo*	Y-79, WERI-RB1	Wang et al. (2020f)
circ-E2F3	Up	miR-204-5p	qRT-PCR	Increased proliferation, migration, invasion, inhibited apoptosis	Human, *in vitro*, *in vivo*	ARPE-19, Y79SO-RB50, WERI-RB1	Huang et al. (2021)
circ_0000527	Up	miR-646	qRT-PCR	Promoted viability, migration, invasion, and RB progression	Human, *in vitro*	SO-Rb50, WERI-RB1, Y79, HXO-RB44, ARPE-19	Chen et al. (2020)
	Up	miR-98-5p	qRT-PCR	Increased proliferation, migration, invasion, decreased apoptosis	*In vivo*, *in vitro*, human	hTERT-RPE1, Y79, WERI-RB1, SO-RB50, ARPE19	Yu et al. (2021)
	Up	miR-1236-3p	qRT-PCR	Promoted proliferation, migration, invasion and angiogenesis, suppressed apoptosis	*In vitro*, *in vivo*, human	ARPE-19, Y79, SO-Rb50	Liang et al. (2022b)
	Up	miR-27a-3p	qPCR	Promoted proliferation, inhibited apoptosis	*In vivo*, *in vitro*, human	Y79, WERI-RB1, ARPE-19	Zuo et al. (2022)
circ-FAM158A	Up	miR-138-5p	qRT-PCR	Enhanced proliferation and metastasis, inhibited apoptosis	*In vivo*, *in vitro*, human	HRA, WERI-RB1, Y79, HXO-RB44, SO-RB50	Zheng et al. (2021)
circ_0099198	Up	miR-1287	RT-qPCR	Enhanced proliferation and metastasis, reduced cell cycle arrest and apoptosis	*In vivo*, *in vitro*	Y79, WERI-RB1, ARPE-19, So-RB50, So-RB70	Jiang et al. (2021b)
circ_0000034	Up	miR-361-3p	qRT-PCR	Increased proliferation, viability, EMT, migration and invasion, suppressed apoptosis	Human, *in vitro*, *in vivo*	ARPE-19, Y79, WERI-RB1, SO-RB50	Jiang et al. (2021a)
	Up	miR-361-3p	qRT-PCR	Promoted proliferation and invasion	Human, *in vitro*	ARPE‐19, Y79, SO-Rb50, WERI-RB1	Sun et al. (2020b)
	Up	miR-361-3p	qRT-PCR	Promoted proliferation, migration, invasion, autophagy and tumor growth, suppressed apoptosis	*In vivo*, *in vitro*, human	Y79, WERI-RB1, SO-Rb50, HXORB44, ARPE-19	Liu et al. (2020c)
circMKLN1	Down	miR-425-5p	qRT-PCR	Inhibited proliferation, migration and invasion	Human, *In vivo*, *in vitro*	ARPE-19, Y79, WERI-RB1	Xu et al. (2021f)
circCUL2	Down	miR-214-5p	qRT-PCR	Inhibited proliferation, invasion and migration	Human, *in vitro*	ARPE-19, Y79, SO-Rb50	Zhang et al. (2022a)
circ_ODC1	Up	miR-422a	RT-qPCR	Promoted proliferation	*In vitro*, Iv vivo, human	ARPE‐19, Y79, WERI‐RB1, SO‐Rb50, HXO‐RB44	Du et al. (2020)
circ_0075804	Up	miR-138-5p	RT-qPCR	Progressed the reproduction, migration and invasion, suppressed the apoptosis	*In vivo*, *in vitro*, human	−	Zhang et al. (2022b)
hsa_circ_0007534	Up	miR-214-3p	qRT-PCR	Promoted viability and tumor formation	Human, *in vivo*, *in vitro*	Y-79, ARPE-19	Lv et al. (2022)

^1^ Human; RB samples obtained from patients.

## 5 Exosomal non-coding RNAs and retinoblastoma

Exosomes are nanosized vesicles 40–100 nm in diameter, which are released by many types of cells and can carry various biomolecules, such as lipids, proteins, mRNAs, and microRNAs. These extracellular vesicles participate in signaling pathways and in cellular communication between cells (Thébaud and Stewart, 2012; Rashed et al., 2017). Exosomes can fuse with the plasma membrane of recepient cells to release their genetic contents into the cytosol. Tumor cells release exosomes which are involved in various steps of angiogenesis (Feng et al., 2017b), immunosuppression (Chen et al., 2018), and tumor progression (Boyiadzis and Whiteside, 2017). In this content, WERI-RB1 cell-derived exosomes could infiltrate into the RB tumor microenvironment to promote tumor development (Chen et al., 2021c) by an unknown mechanism. However, the microRNAs contained in exosomes are known to exert a powerful effect upon tumor pathogenesis. For example, miR-210 contained inside hepatocellular carcinoma (HCC)-derived exosomes can stimulate angiogenesis by direct inhibition of SMAD4 and STAT6 (Lin et al., 2018). Furthermore, the miRNA-25-3p contained inside colorectal cancer (CRC)-derived exosomes can promote vascular leakage and increase the CRC metastatic phenotype (Zeng et al., 2018).

Krüppel-like factor 2 (KLF2) is an important member of the KLF family, a subclass of zinc-finger-containing transcription factors with DNA-binding domains, which can regulate tumor proliferation, metastasis, and affect the microenvironment (Tetreault et al., 2013). This molecule could inhibit the VEGFR2 promoter activity leading to reduced angiogenesis (Bhattacharya et al., 2005). KLF2 was downregulated by exosomal miR-25-3p to increase angiogenesis and raise vascular permeability in CRC (Zeng et al., 2018). Interestingly, KLF2 was found to be downregulated in breast (Zhang et al., 2015c), colorectal (Wang et al., 2017g), and lung cancer (Li et al., 2016c). Despite its downregulation in multiple cancer types with different stages and grades, KLF2 is highly expressed in normal epithelial cells (Wang et al., 2005).

Chen et al., identified the function and potential mechanism of exosomes released from WERI-RB1cells in RB tumor angiogenesis using biochemical approaches and animal experiments (Chen et al., 2021d). The *in vitro* findings showed that the exosomes were possibly taken up by human vascular endothelial cells (HUVECs). These exosomes led to increased survival and an inflammatory response in HUVECs by upregulation of genes, such as VCAM1, ICAM1, IL-1, IL-6, IL-8, and MCP-1. Additionally, cell migration and tube formation were significantly increased in HUVECs treated with RB cell-derived exosomes. Furthermore, *in vivo* data showed that tumors could absorb exosomes which were injected near the tumors. Compared to control tissues, the number of blood vessels and endothelial cells was increased in tumor tissues incubated with exosomes. The mechanism of stimulation of angiogenesis by the RB-derived exosomes was propsed to be miR-92a-3p contained within the exosomes. When HUVECs were treated with these exosomes they showed an increased expression of this miR. Treatment of HUVECs with a miR-92a-3p inhibitor significantly abrogated the effect of exosomes on tube formation and migration, as well as downregulation of the angiogenesis-related genes. The opposite results were obtained after treatment of HUVECs with a miR-92a-3p mimic. Bioinformatics analysis showed that KLF2 mRNA might be targeted by miR-92a-3p, which was confirmed both *in vitro* and *in vivo*. Therefore exosomal miR-92a-3p could be a promising therapeutic option for RB (Chen et al., 2021d).

Plousiou et al., examined the regulatory role of miRNAs in RB using a zebrafish model, (Plousiou et al., 2022). They first showed that co-culture of monocytes with RB cell lines significantly reduced proliferation, and the monocyte conditioned medium was added to RB cells to investigate the effect of the supernatant on RB progression. The found that miR-142-3p was upregulated in RB cells as well as in the medium used for their culture, compared with controls. Next they showed that monocytes secreted exosomes, which carried miR-142-3p into the co-culture medium and were then taken up by RB cells, leading to cell cycle arrest and inhibition of proliferation. They proposed that miR-142-3p could target the mRNA for TGFβR1 (transforming growth factor β receptor). Therefore, exosomal miR-142-3p could be a new miR-based treatment strategy to control RB tumor growth (Table 4) (Plousiou et al., 2022).

**TABLE 4:** The role of exosomal ncRNAs in retinoblastoma.

Cargo	Target	Detection method	Effect	Model	Type of cell line	Reference
miR-92a-3p	KLF2	RT-PCR	Promoted migration and tube formation, and increased expression of angiogenesis-related genes	*In vitro*, *in vivo*	WERI-RB1	Chen et al. (2021d)
miR-92a, miR-20a, miR-129a, miR-17	−	RT-qPCR	Increased viability and proliferation, inhibited the antitumor activity of macrophages and innate immunity, promoted RB tumor growth	*In vitro*, *in vivo*	WERI-RB1	Chen et al. (2021d)
miR-142-3p	TGFβR1	qRT-PCR	Inhibited proliferation, induced cell cycle arrest	*In vitro*, *in vivo*	CHLA-215	Plousiou et al. (2022)

## 6 Conclusion

The importance of ncRNAs in affecting many normal physiological processes in humans, as well as pathological pathways is becoming increasingly appreciated. These include cell differentiation, proliferation, apoptosis, and migration. It has been widely accepted that deregulation of ncRNAs is linked to various diseases, particularly different cancers. RB is no exception, and several ncRNAs have now been identified as either tumor suppressor genes or tumor promoter genes. However it is known that several ncRNAs can have opposite functions across various cancer types, and even within the same tumor depending on stage, underlining the importance of characterizing the particular ncRNAs, and their mechanisms of action. Future research should focus on identifying the regulatory roles of ncRNAs and their related mechanisms in RB. Furthermore, the clinical applications of ncRNAs are yet to be evaluated. In addition to their value as prognostic or diagnostic biomarkers of disease, more studies are warranted to assess whether ncRNAs can be used in therapeutic regimens for RB, and whether exosomal ncRNAs may be more useful.
